# Biosensor-Based Multimodal Deep Human Locomotion Decoding via Internet of Healthcare Things

**DOI:** 10.3390/mi14122204

**Published:** 2023-12-03

**Authors:** Madiha Javeed, Maha Abdelhaq, Asaad Algarni, Ahmad Jalal

**Affiliations:** 1 Department of Computer Science, Air University, Islamabad 44000, Pakistan; ahmadjalal@mail.au.edu.pk; 2 Department of Information Technology, College of Computer and Information Sciences, Princess Nourah bint Abdulrahman University, P.O. Box 84428, Riyadh 11671, Saudi Arabia; 3 Department of Computer Sciences, Faculty of Computing and Information Technology, Northern Border University, Rafha 91911, Saudi Arabia; asaad.algarni@nbu.edu.sa

**Keywords:** human activity recognition, Internet of Healthcare Things, locomotion prediction, multimodal systems, recurrent neural network, RGB, wearable sensors

## Abstract

Multiple Internet of Healthcare Things (IoHT)-based devices have been utilized as sensing methodologies for human locomotion decoding to aid in applications related to e-healthcare. Different measurement conditions affect the daily routine monitoring, including the sensor type, wearing style, data retrieval method, and processing model. Currently, several models are present in this domain that include a variety of techniques for pre-processing, descriptor extraction, and reduction, along with the classification of data captured from multiple sensors. However, such models consisting of multiple subject-based data using different techniques may degrade the accuracy rate of locomotion decoding. Therefore, this study proposes a deep neural network model that not only applies the state-of-the-art Quaternion-based filtration technique for motion and ambient data along with background subtraction and skeleton modeling for video-based data, but also learns important descriptors from novel graph-based representations and Gaussian Markov random-field mechanisms. Due to the non-linear nature of data, these descriptors are further utilized to extract the codebook via the Gaussian mixture regression model. Furthermore, the codebook is provided to the recurrent neural network to classify the activities for the locomotion-decoding system. We show the validity of the proposed model across two publicly available data sampling strategies, namely, the HWU-USP and LARa datasets. The proposed model is significantly improved over previous systems, as it achieved 82.22% and 82.50% for the HWU-USP and LARa datasets, respectively. The proposed IoHT-based locomotion-decoding model is useful for unobtrusive human activity recognition over extended periods in e-healthcare facilities.

## 1. Introduction

Recent trends in the Internet of Healthcare Things (IoHT) have boosted wearable and visual-technology-based human locomotion decoding. This boost converts the healthcare industry from cure to prevention [1,2,3,4]. Various IoHT devices are available for healthcare and research, including smart devices, inertial units, and cameras. Data from such IoHT devices have been extracted, processed, and analyzed for human locomotion decoding. For ambient assisted living, sensor-based data have been used to support and supervise people, also known as human activity recognition (HAR) [5,6,7]. Applications of such HAR systems include injury recognition, medical analysis, long-term or short-term care, health monitoring, and independent quality of life [8,9,10,11,12].

These HAR systems can use machine learning or deep learning techniques to decode the activities of daily living by extracting data from motion, ambient, or vision-based sensors [13,14,15,16]. Modern smart devices manipulate the data and thus cannot be utilized for locomotion decoding [17,18,19,20]. Some HAR systems have less efficiency due to errors induced by the data acquisition that must be resolved using a robust filter [21,22,23]. Exiting feature extraction methods cannot perform well for HAR systems and provide less efficient results [24,25,26,27,28]. Therefore, a multimodal sensor-based human-locomotion-decoding (HLD) system consisting of motion, ambient, and vision sensors is proposed in this paper. The key contributions of this research are as follows:

- An innovative multimodal system for locomotion decoding via multiple sensors fused to enhance the HAR performance [29,30,31];
- The effective and novel filtration of the inertial sensor data [32,33,34] by using a proposed state-of-the-art Quaternion-based filter;
- A novel approach to filtering the ambient-based data that includes infrared cameras and switches attached to the environment;
- Hand-crafted contemporary descriptor extraction methods [35,36,37,38] are proposed and applied to acquire related descriptors [39,40,41,42] using novel techniques;
- Efficient ambient sensor descriptor extraction based on a unique and novel graph representation;
- The proficient recognition of activities [43,44,45,46] for locomotion decoding via detection through a recurrent neural network (RNN).

Section 2 explains the sensor-based activity recognition systems presented in the literature. Next, Section 3 details the proposed locomotion-decoding system for the IoHT industry [47,48,49,50,51]. The experiments performed over the selected datasets using the proposed method and their results, along with a comparison of the baseline system and previous state-of-the-art models, are discussed in Section 4. The conclusion of the whole paper is presented in Section 5.

## 2. Related Work

Locomotion decoding with a combination of IoHT-based sensors can be utilized for different applications [52,53,54,55], including the execution and tagging of data, which associates the meanings of sensor data interpretations by using symbols. A single sensor is not enough to provide the semantic meaning of a situation. Therefore, multimodal sensor-based systems serve this purpose. For this resolution, multiple systems have been proposed in history to evaluate the effectiveness, completeness, and reliability of such sensor-based decoding systems.

### 2.1. Sensor-Based Locomotion Decoding

In [56], Franco et al. propose a multimodal system for locomotor activity recognition. They used RGB video and other sensors for data acquisition. Histograms of oriented gradient (HOG) descriptors and skeleton-based information were extracted from the RGB data frames to capture the most prominent body postures. For the activity classification, a voting system was defined to obtain votes from support vector machine (SVM) and random forest classifiers. However, the proposed system could not achieve higher results due to the absence of a filtration technique for the data. Another system is proposed in [57] that collects motion sensor data. Next, data are processed using a linear interpolation filter and segmentation. Features are extracted using four different extraction techniques and normalized using the z-score function. Then, features are selected via correlation and evolutionary search algorithms. Further, the class imbalance is removed using the synthetic minority over-sampling method. Features are fused, and multi-view stacking is utilized to classify humans.

### 2.2. Multimodal Locomotion Decoding

In [58], the authors propose a robust human activity recognition method. They used multimodal data based on wearable inertial and RGB-D sensors. The inertial data were pre-processed using magnitude computation and noise removal techniques, and dense HOGs were extracted from video data. Time domain features are extracted from inertial signals, and bag-of-words encoding is utilized for video frame sequences. Furthermore, the features are fused, and K-nearest-neighbor and support vector machines are used for the human activity classification. 

A long short-term memory (LSTM) network-based system is proposed in [59]. To recognize activities of daily living, the authors used a deep learning model via data acquired from real-world and synthetic environments. The sensors were attached to the wrists, ankles, and waist to detect activities, including eating and driving. Each sensor’s accuracy was observed to elaborate the custom weights for each sensor fusion. This study recommended using one sensor on the upper body parts and one sensor on the lower body parts to obtain reasonable results. However, due to the restricted data used and limited weight learning in the system, the method cannot adapt to changes over time.

In [60], a system of Marfusion based on a convolutional neural network (CNN) and attention mechanism is proposed. Features are extracted from multimodal sensors and a set of CNNs is utilized for each sensor. Next, a dot-product, scaled, self-attention process is applied to give weight to each sensor. Then, CNN and attention-based modules are utilized for feature fusion with different parameters. Further, fully connected batch normalization, dropout, ReLU, and softmax layers are used for the classification via the obtainment of the probabilities for different activities. The proposed system gave an acceptable performance but experimented with limited human locomotion. Therefore, the results are not robust for real-time environments.

## 3. Materials and Methods

**Figure 1:**
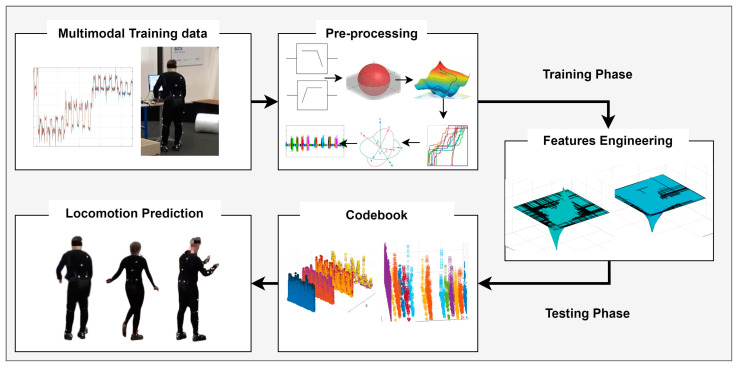
Architecture diagram of proposed IoHT-based human-locomotion-decoding system.

The proposed locomotion-decoding architecture is described in Figure 1. The input data for the proposed IoHT-based system were taken from two publicly available datasets named Logistic Activity Recognition Challenge (LARa) [61] and Heriot-Watt University/University of Sao Paulo (HWU-USP) [62], which are present in the form of time series in a time segment of size W from S sensors. Sensors of three types were used: physical signals {*pi*}, ambient signals {*pa*}, and visual frame sequences {*pv*}. Algorithm 1 demonstrates the complete IoHT-based HLD system. The input {*pi*, *pa*, *pv*} from the *S* sensors was pre-processed for each time segment of a *W* size. Next, the descriptors were extracted and optimized {Vi*, Ki*, Si*, Ai*} for each *W* segment. Further, the descriptors were trained by using an RNN and tested the remaining descriptors to recognize activities {A*} to decode human locomotion. All these phases of the IoHT-based HLD system are further explained in the next subsections.


**Algorithm 1 HLD Algorithm**
**Input:** physical IMU signals {*p_i_*}, ambient signals {*p_a_*}, visual frame sequences {*p_v_*};
**Output:** recognized activities {A*};Pre-process {*p_i_*, *p_a_*, *p_v_*} for each segment *W* in *Module I*;Extract descriptors {V_i_*, K_i_*, S_i_*, A_i_*} for *W* in *Module II*;Optimize descriptors for *W* in *Module III*;Train descriptors over classifier to obtain *f*(*X*,*θ*);Test remaining descriprtors to obtain {*θ*,θ*};Recognize activities {A*};

### 3.1. Pre-Processing Motion and Ambient Data

A novel quaternion-based filter is proposed in this study to pre-process the physical-motion [63] and ambient data from the sensor inertial measurement units (IMUs). The signals are clarified via low- and high-pass Butterworth filters [64,65] for further processing. Next, the signals are normalized using the Euclidean distance [66,67]:

(1) $$Norm=\sqrt{LPF_{1}+LPF_{2}+LPF_{3}}+\sqrt{HPF_{1}+HPF_{2}+HPF_{3}}\;$$

where *LPF*_1_, *LPF*_2_, and *LPF*_3_ denote the filtered values for the x-, y-, and z-axes via the Butterworth filter, respectively. *HPF*_1_, *HPF*_2_, and *HPF*_3_ represent the filtered values of the x-, y-, and z-axes through the Butterworth filter, respectively.

Then, for the accelerometer signals, gravity from a stationary activity, such as lying down, is extracted as the minimum gravity ($g_{m}$) and average gravity ($g_{a}$). Then, the gravitational error ($g_{e}$) [68,69] is removed from the accelerometer signals, giving more accurate and error-free signals for further processing. Similarly, the earth’s magnetic field is used to remove the magnetic errors from magnetometer signals [70,71].

**Figure 2:**
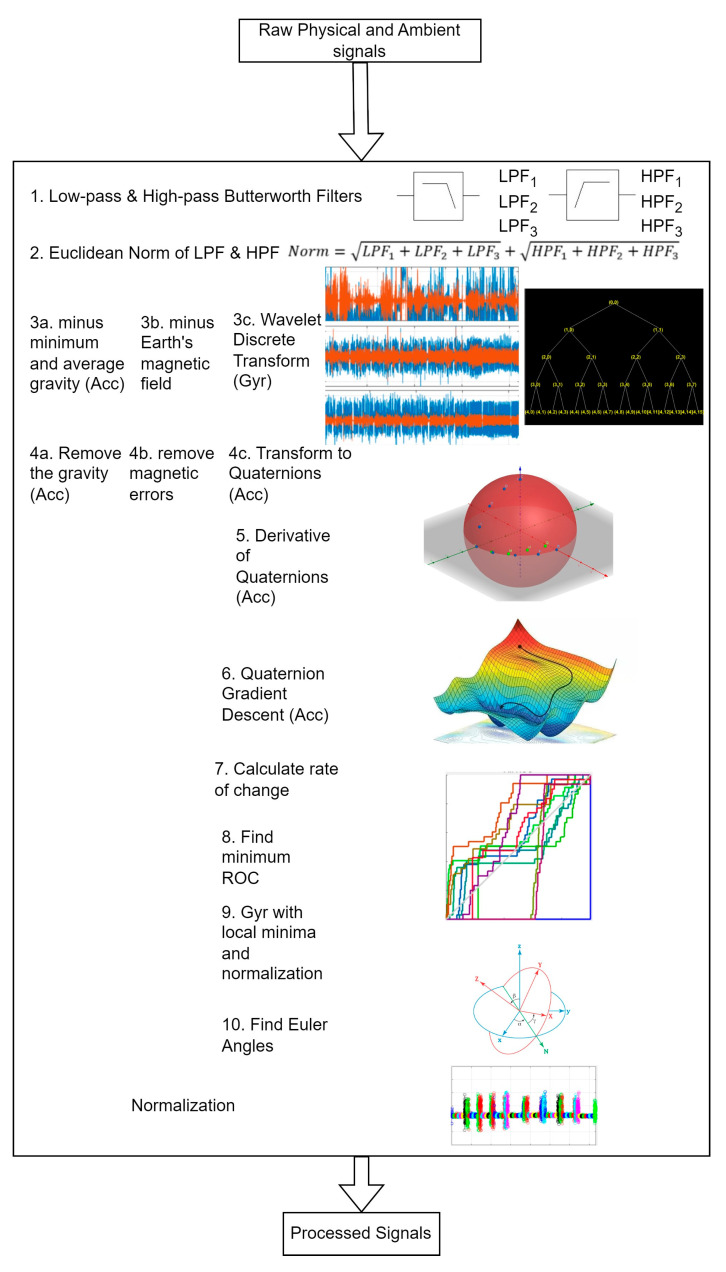
Pre-processing module proposed for physical-motion and ambient data.

After normalization, discrete wavelet transform [72] is applied to the gyroscope signals to transform them into quaternions in order to avoid the gimbal lock problem. Later, the derivative of the quaternions is considered, and gradient descent is applied to attain the minimum rate of change. Further, a local minimum [73] is selected, and the gyroscope signals are normalized using the Euler angles:

(2) $$Axz=atan2\left(z,x\right),$$

(3) $$Ayz=atan2\left(z,y\right),$$

(4) $$Axy=atan2\left(y,x\right),$$

where Axz, Ayz, and Axy are the Euler angles. Lastly, all three pre-processed signals are normalized together. Figure 2 explains the pre-processing step for the physical-motion module in detail.

### 3.2. Pre-Processing Visual Data

**Figure 3:**
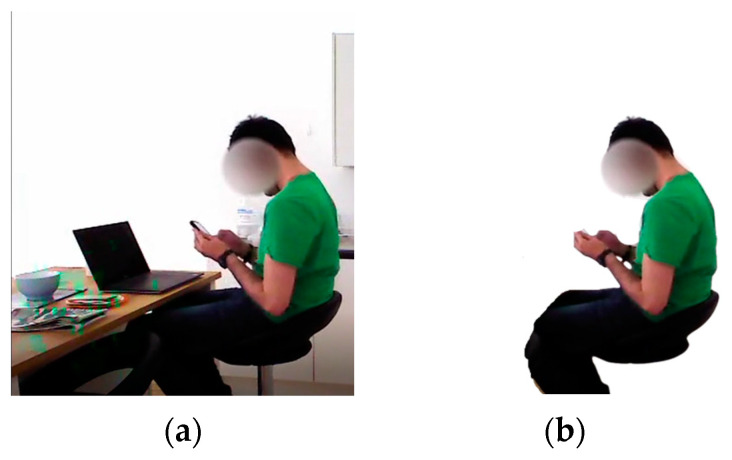
(**a**) Before background deduction and (**b**) after background deduction of a frame sequence from HWU-USP dataset.

For the pre-processing, videos from both datasets were converted into frame sequences. A delta of 50 was chosen to restrict the number of pre-processing sequences to avoid redundant data processing. Next, we retrieved a background image from both data sequences. Then, the background was removed by subtracting the background image from the original frame sequences [74,75]. The background subtraction from the original image sequence is displayed in Figure 3. Discrete wavelet transform was used over the frame sequences to reduce the noise present.

**Figure 4:**
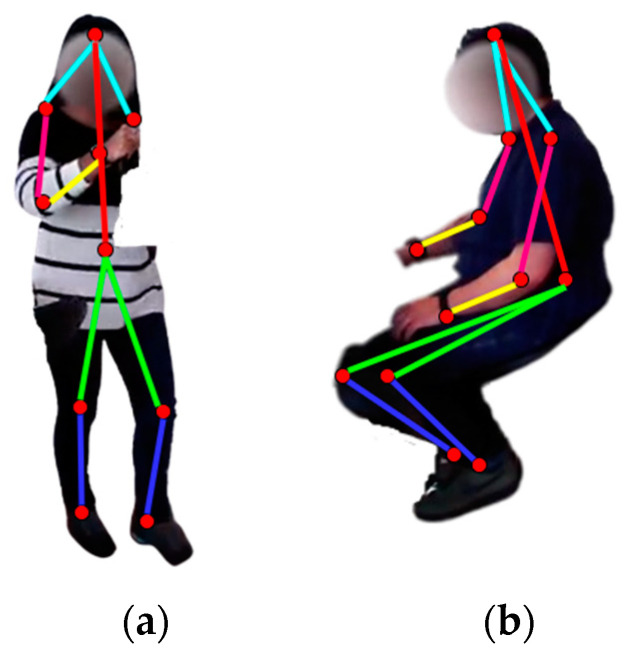
Skeleton point decoding from frame sequences of (**a**) drinking tea and (**b**) reading a newspaper.

Skeleton modeling was performed through blob and centroid techniques for human detection in the frame sequences. First, the blobs were defined from the human movable parts, which was followed by taking the centroids and deciding on five types of skeleton body points—head, shoulders, elbows, wrists, torso, knees, and ankles [76]. Figure 4 shows the skeleton points extracted for drinking tea and reading a newspaper. 

### 3.3. Data Segmentation

**Figure 5:**
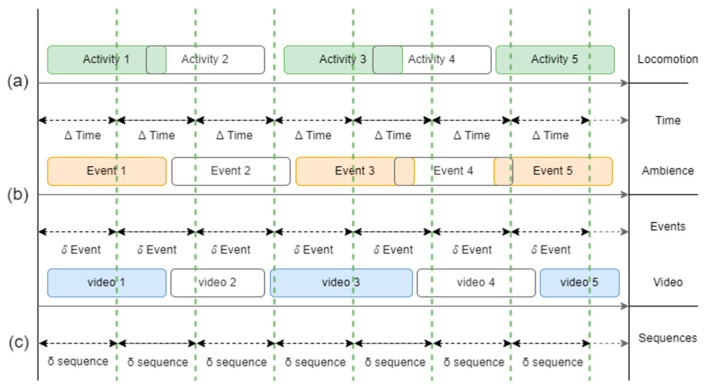
Segmentation for (**a**) motion, (**b**) ambient, and (**c**) visual data.

Next, to deal with the dimensions of the datasets, this study segmented the motion and ambient pre-processed data into overlapped [77] and time-based [78] segments, whereas the vision-based data were segmented through event-based segments. For all three types of data $\left\{p_{i}{}^{*},\;p_{a}{}^{*},\;p_{v}{}^{*}\right\}$, Figure 5 shows the segmentation process by using manifold locomotion activities.

### 3.4. Motion Descriptor Extraction

The pre-processed and segmented motion-based data were further provided to two different techniques for the descriptor extraction, including Gaussian Markov random field (GMRF) and a novel contribution in the form of a multisynchrosqueezing transform (MSST)-based spatial–temporal graph.

**Figure 6:**
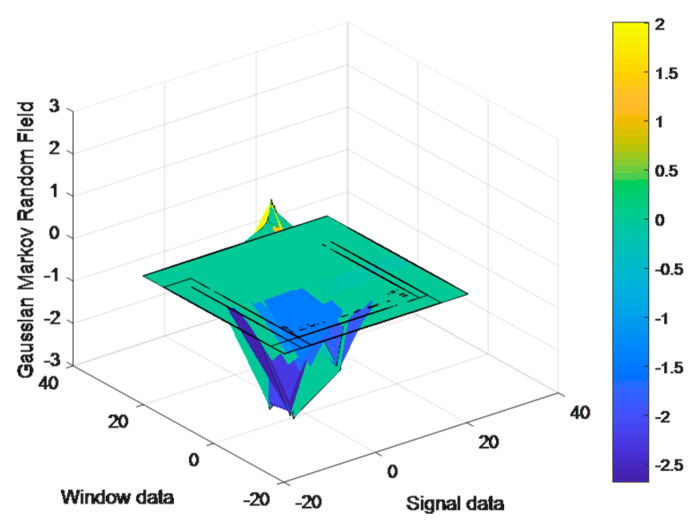
Results of GMRF application on HWU-USP dataset.

GMRF can take multidimensional data, and a stochastic process becomes Gaussian when all its distributions are Gaussian-normalized [79]. Equations (5) and (6) show the expectation function (${\tilde{\mu }}_{t}$) and covariance function ($\tilde{\sum }s,t$) using *s* samples and *t* times. Figure 6 presents the results for the GMRF for a window of kinematic physical data on HWU-USP.

(5) $${\tilde{\mu }}_{t}=E{\tilde{X}}_{t},$$

(6) $$\tilde{\sum }s,t=\mathrm{cov}\left({\tilde{X}}_{s},{\tilde{X}}_{t}\right).$$

**Figure 7:**
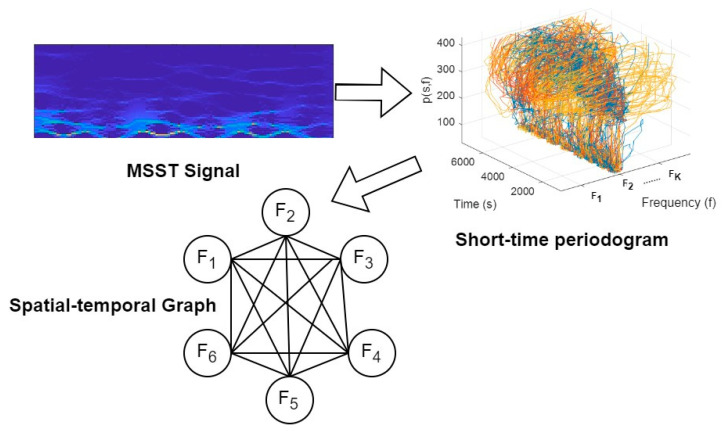
Process of constructing novel spatial–temporal graph from MSST.

MSST represents multiple synchrosqueezing transforms iteratively [80] and is calculated as follows:

(7) $$Ts^{\left[M\right]}\left(t,\gamma \right)=\int_{-\infty }^{+\infty }Ts^{M-1}\left(t,\gamma \right)\delta \left(\gamma -\hat{\omega }\left(t,\omega \right)\right)d\omega ,$$

where M gives the iteration number ≤2 and $Ts^{\left[M\right]}\left(t,\gamma \right)$ is the spread time–frequency coefficient. The short-time periodogram is further calculated as follows:

(8) $$p\left(s,f\right)=\frac{1}{T}\;|\;Y\left(s,f\right){|}^{2}$$

where $p\left(s,f\right)$ is the result of frequency (f) and time (s). T shows the window length. Further, the spatial–temporal graph was constructed using six nodes or frequencies. Figure 7 shows the novel spatial–temporal graph extracted from MSST for a random static pattern.

### 3.5. Ambient Descriptor Extraction

**Figure 8:**
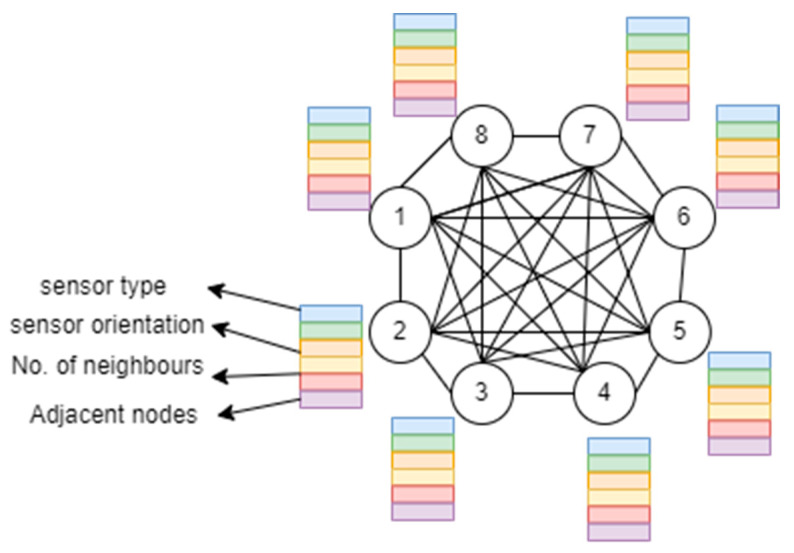
Proposed novel graph-based ambient feature extraction.

A graph-based representation has been proposed as a novel descriptor extraction for ambient sensor pre-processing [81]. For each sensor attached to the ambient, a graph (*R*) is produced using a descriptor matrix (*M*) and adjacency matrix (*K*) given by the following:

(9) $$R=\left(M,K\right)$$

where M is the descriptor matrix consisting of the sensor type, number of neighbors, and sensor orientation. K contains the number of adjacent sensors for each node and the names of neighboring sensors. Figure 8 presents the details of the proposed graph-based ambient descriptors.

### 3.6. Vision Descriptor Extraction

**Figure 9:**
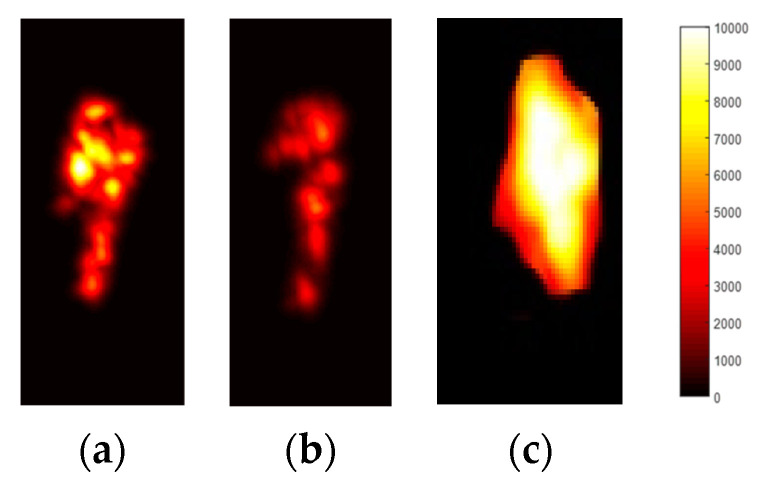
Thermal heat map extracted for activities including (**a**) drinking tea, (**b**) opening a drawer, and (**c**) reading a newspaper.

In thermal descriptors, the movement from one frame to another is captured in the form of thermal maps. More movement is described using higher heat values in yellow, and less movement is shown using red or black [82]. In Equation (10), x represents a one-dimensional vector comprising the extracted values, i represents the index value, and R denotes the RGB value. Figure 9 presents the heat map for the full-body frame sequence.

(10) $$TM\left(x\right)=\sum_{i=0}^{k}ln\;R\left(i\right).\;$$

**Figure 10:**
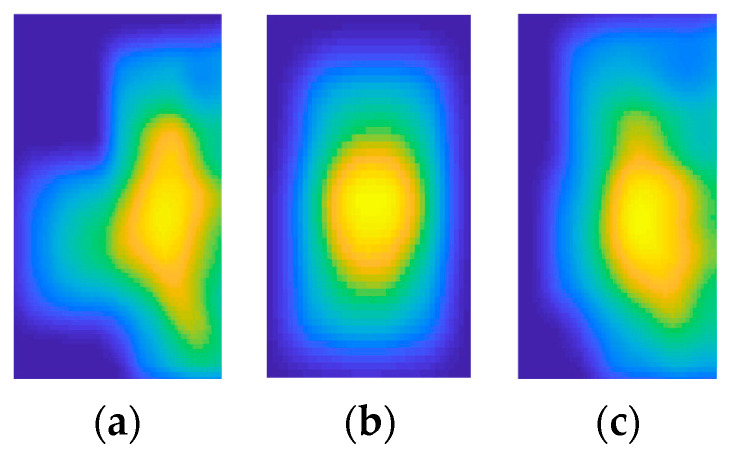
Results of saliency maps applied over full-body frame sequences for (**a**) drinking tea, (**b**) opening a drawer, and (**c**) reading a newspaper.

The full-body descriptor extraction method for visual data utilized is called the saliency map (SM) approach. It is computationally expensive to process an entire frame simultaneously; therefore, the SM approach suggests sequentially looking at or fixating on the salient locations of a frame. The fixated region is analyzed, and then attention is redirected to other salient regions using saccade movements requiring more focus [83]. The SM approach is a successful and biologically plausible technique for modeling visual attention. The generalized Gaussian distribution shown in Equation (11) is used to model each of these:

(11) $$P\left(f_{i}\right)=\frac{\theta_{i}}{2\sigma_{i}\gamma \left(\theta_{i}^{-1}\right)}\exp\left(-|\frac{f_{i}}{\sigma_{i}}{|}^{\theta_{i}}\right),$$

where $\theta_{i}>0$ is the shape parameter, $\sigma_{i}>0$ provides the scale parameter, and γ gives the gamma function. Figure 10 presents the results of SMs applied over a full-body frame sequence.

**Figure 11:**
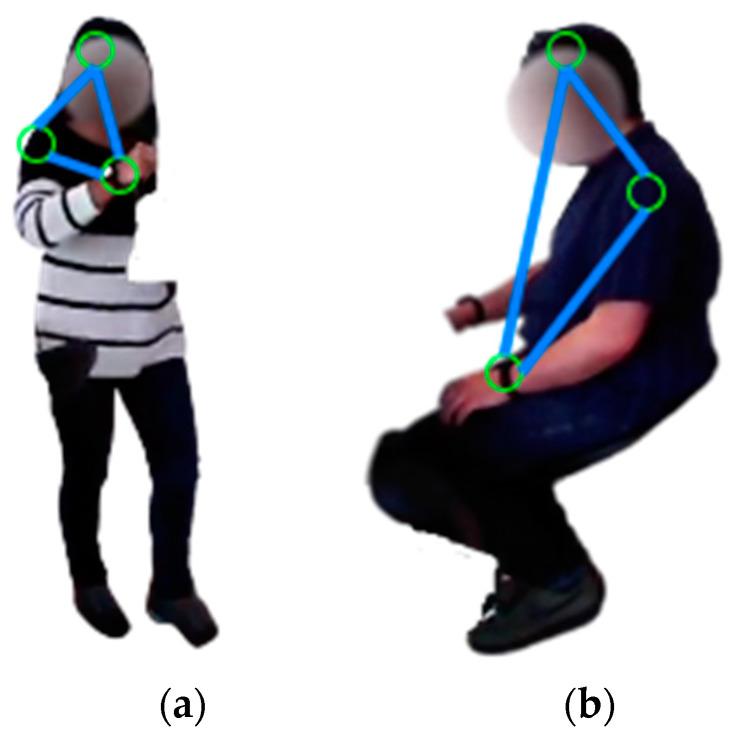
The triangular shape is formed by combining human skeleton body points for (**a**) drinking tea and (**b**) reading a newspaper.

The orientation descriptor technique is the first descriptor extraction technique for the skeleton body points. Five skeleton body points are used to make triangles and obtain angles from them. The tangent angle in Equation (12) is measured between the three sides of each triangle [84]:

(12) $$\tan\;\theta =\frac{u\cdot v}{\left|u\right|\left|v\right|},$$

where u·v is the dot product of vectors u and v that are any two sides of a triangle. Figure 11 demonstrates the examples of triangles formed by combining two human skeleton body points in some activities, such as drinking tea and reading a newspaper.

**Figure 12:**
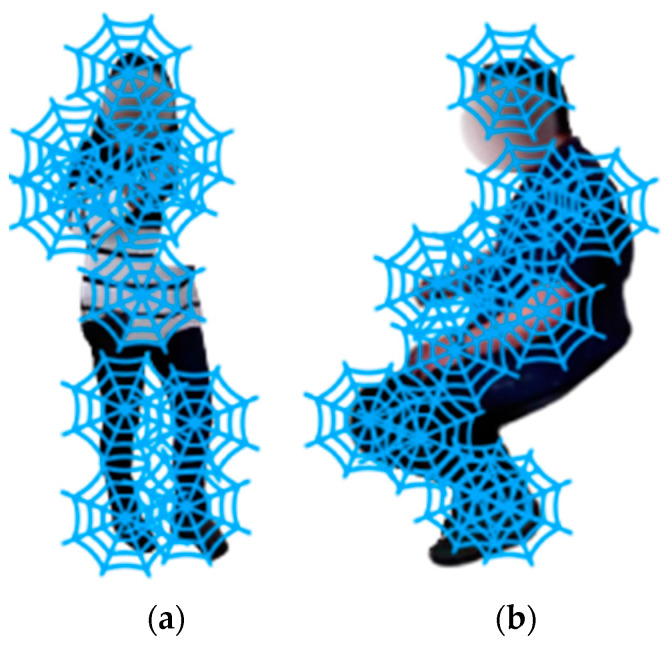
Spiderweb applied for (**a**) drinking tea and (**b**) reading a newspaper over HWU-USP dataset.

The second descriptor extraction technique used for the skeleton body points is the spider local image feature (SLIF) technique. A spiderweb representation emulates the skeleton body point nodes as web intersection points in a frame sequence [85,86]. The position of each node (n,m) is denoted by a set of two-dimensional coordinates, as follows:

(13) $$x_{n,m}=\left(\frac{m\cdot cos\left(\frac{2\pi n}{N}\right)}{M},\frac{m\cdot sin\left(\frac{2\pi n}{N}\right)}{M}\right),$$

where the first and second terms represent the horizontal and vertical coordinates, respectively. For a set of previously defined skeleton body points, the SLIFs are extracted by selectively extracting pixel information from around the neighborhood of each point and applying a spiderweb over the point. Figure 12 shows a spiderweb applied over two sample frame sequences.

### 3.7. Codebook Generation

A Gaussian mixture model (GMM) codebook is used to encode the descriptors extracted from previous subsections. An expectation maximization (EM) algorithm is used in the GMM to present complex descriptors. This algorithm approximates the parameter set (*Θ*) and aids in calculating the maximum likelihood through an initial parameter set (*Θ*1), and then continuously applies the *E* and *M* steps. Then, it produces {*Θ*1, *Θ*2, …, *Θm*, …} and both *E* and *M* steps as follows:

(14) $$\gamma^{m}\;(z_{k}^{j}\;|\;x_{j},\;\Theta^{m})=\frac{\omega_{k}^{m}\;f(x_{j}|\mu_{k}^{m},\;\;\;\sum_{k}^{m})}{\sum_{i=1}^{K}\omega^{m}\;f(x_{j}|\;\mu_{i}^{m},\;\;\;\sum_{i}^{m})},$$

(15) $$\sum_{k}^{m-1}=\frac{\sum_{j=1}^{N}\gamma^{m}(z_{k}^{j}|x_{j},\;\;\Theta^{m})\left(x_{j}-\mu_{k}^{m+1}\right){\left(x_{j}-\mu_{k}^{m+1}\right)}^{T}\;}{\sum_{j=1}^{N}\gamma^{m}(z_{k}^{j}|x_{j},\;\;\Theta^{m})\;}.$$

where $\gamma^{m}\;(z_{k}^{j}\;|\;x_{j},{\;\Theta }^{m})$ gives the probability of the *jth* example and the *kth* Gaussian at the *mth* iteration with weights ($\omega_{k}^{m}$), means ($\mu_{k}^{m}$), and covariance ($\overset{m}{\underset{k}{\sum }}$) values. Similarly, a single generalized signal is extracted from the set of descriptors given using Gaussian mixture regression (GMR). Henceforth, a smooth signal via regression can be taken out by coding the temporal signal features [87] through a mixture of Gaussians. Each vector of the signals’ GMM is taken as the input (xI) and output (xO) using GMR via this method.

### 3.8. Locomotion Decoding

**Figure 13:**
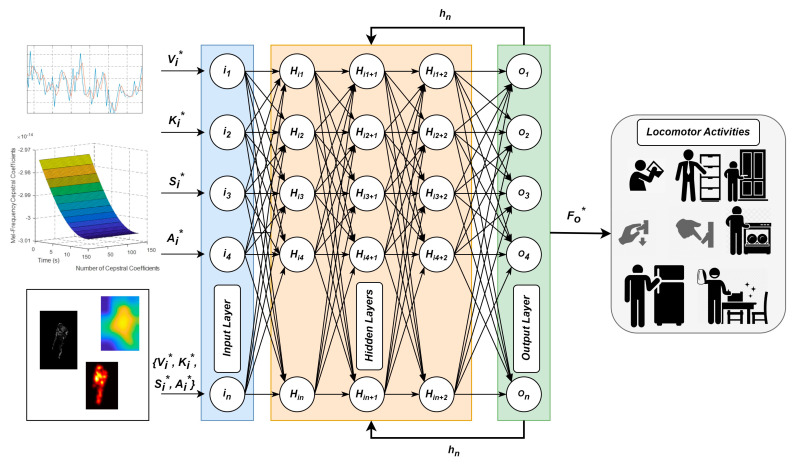
RNN incorporated into the proposed IoHT-based HLD system.

A simple feedforward neural network poorly handles the sequence of data. It never forms a cycle between two hidden layers, and information always flows in one direction, never going back. It comprises an input layer, a hidden layer, and an output layer. An RNN [88] also contains these three layers, but it focuses on considering the current state along with the previous state in the form of output from the previously hidden layer via memory. Thus, the current state and previous state are used to produce output for the next time step, as shown in Figure 13. An activation function is also used to calculate the current state; we used tan *h* as the activation function. Due to the input pattern change, the RNN performs better by incorporating backpropagation.

## 4. Performance Evaluation

In order to evaluate the IoHT-based HLD system, the following datasets and evaluation criteria were used.

### 4.1. Dataset Descriptions

**Figure 14:**
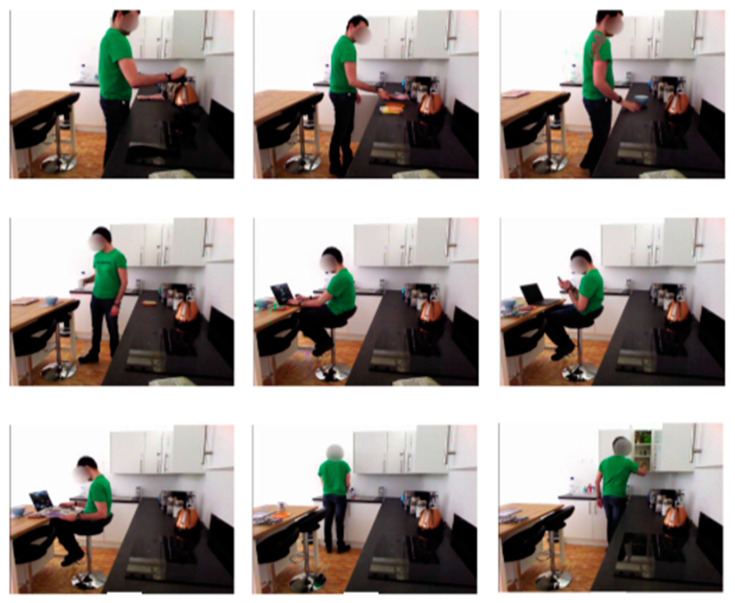
Activities performed by subject in HWU-USP dataset [61].

**Figure 15:**
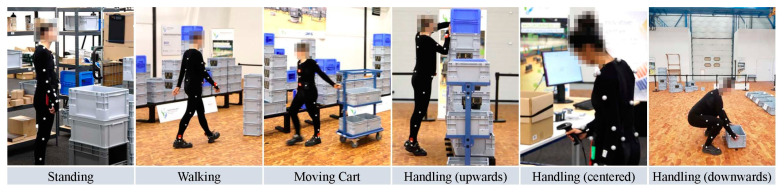
Sample frame sequences from the LARa dataset [62].

Several publicly available datasets are present for human locomotion decoding via activity recognition. However, they can be different in terms of the number of subjects, number of activities performed, environmental setup, number of sensors, type of sensors, and sampling rates. In the proposed IoHT-based HLD system, we used two publicly available datasets, HWU-USP [62] and LARa [61], captured in diverse environmental setups and three different sensor modalities to make the system more robust. A 10-fold cross-validation technique was utilized to evaluate the proposed system. The following sections give details on the datasets mentioned above:

HWU-USP: A dataset recorded in a “living-lab” was selected for this study. It contains recordings from binary switches, PIR sensors, RGBD cameras installed over a robot, and IMU devices. The camera color is VGA 640 × 480 at 25 fps. A total of 16 participants performed nine activities with 144 instances with an average length of 48 s [62]. The participants were voluntary and healthy with neither functional nor visual impairments. The dataset contains activities of daily living with either periodical patterns or long-term dependencies and, hence, it is different from other multimodal environments. A variety of activities have been performed, such as *making a cup of tea*, *making a sandwich*, *making a bowl of cereal*, *setting the table*, *using a laptop*, *using a phone*, *reading a newspaper*, *cleaning the dishes*, and *tidying the kitchen*. Figure 14 represents the sample of activities performed by one of the participants in the HWU-USP dataset;

LARa: This dataset consists of an OmoCap system, a VICON system of 38 infrared cameras, three sets of IMU devices, and 30 recordings of 2 min for each of the 14 subjects. A wide range of participants were selected, including both male and female, ranging in age from 22 to 59 years, weighing from 48 to 100 lbs, left- and right-handed, and with heights from 163 to 185 cm. The dataset was recorded in a total of seven sessions of 758 min of recording. Acceleration sensors recorded the locomotion at a rate of 100 Hz [61]. The dataset is unbalanced regarding the annotations due to the complex process. The dataset is based on the activities performed in a logistics-based context. An expert trained the subjects in advance to recordings. A total of eight activities were recorded for each subject, including standing, walking, carting, handling (upwards), handling (centered), handling (downwards), synchronization, and none. Figure 15 gives a few sample frame sequences from the dataset.

### 4.2. Experiment 1: Evaluation Protocol

Evaluation metrics can be used to evaluate the performance of the chosen deep learning classifier, including the accuracy, precision, and *F*1-score [89]. Table 1 shows the evaluation metrics derived from the experimental results. In our study, these metrics were chosen where the accuracy was the ratio between the decoded samples and the total number of samples. The three metrics can be defined as follows:

(16) $$Acc=\frac{TP+TN}{TP+FN+FP+TN},$$

(17) $$rec=\frac{TP}{TP+FN},$$

(18) $$F1-score=\frac{2\times \left(rec\cdot pre\right)}{\left(rec+pre\right)},$$

where TP,TN are the true-positive and true-negative values, FP,FN give the false-positive and false-negative values, and pre is the precision, which can be calculated as follows:

(19) $$pre=\frac{TP}{TP+FP}$$

### 4.3. Experiment 2: Comparison with Baseline HLD Systems

In the first experiment, we tested to highlight the importance of novel techniques introduced in this system. The first novelty is the motion and ambient data filtration technique that can handle sensor signal-based errors, biasness, and drift. The second novelty is ambient and motion descriptor extraction through a graph-based approach that helps extract robust descriptors related to the data type. The comparative results for the proposed IoHT-based HLD system with the first novelty, second novelty, and both together are given in Table 1, along with a comparison of the same system classification through the CNN [90] and LSTM [91].

We used the scikit-learn library to train all three classifiers. We set the learning rate for the CNN to 0.001, and the maximum epoch number was 200. The input layer contained the descriptors extracted. Then, we proposed three convolution layers with the ReLU activation function. Next, the pooling layer was utilized after each convolution layer. A flattened layer was also used to flatten the shape of the layers. Further, a fully connected layer with two hidden layers and a softmax layer were also used to test the trained data through output. For the LSTM, we used the architecture proposed in [92], where an input layer, a few LSTM-based temporal models, a flattened layer, and a fully connected network were used to recognize the ADL. Table 2 shows the confidence levels of extracted skeleton body-points compared to the ground truth values over HWU-USP and LARa datasets.

### 4.4. Experiment 3: Comparison with Other Works Utilizing Filtration and Descriptors

**Figure 16:**
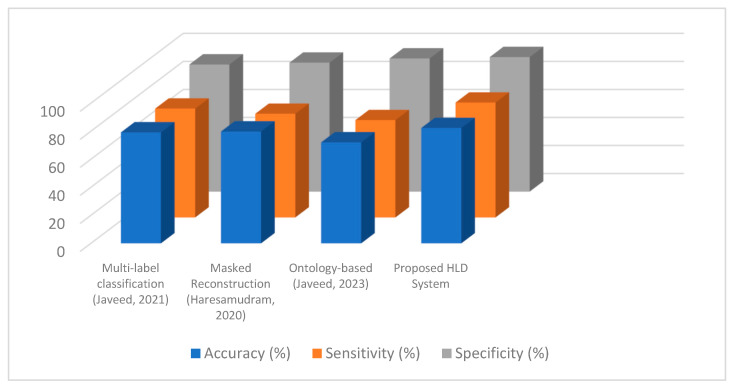
Comparison of previous works [93,94,95] with proposed HLD systems over the two novelties proposed.

This section will focus on comparing the two novelties with the existing techniques by comparing them with the proposed HLD system. Figure 16 compares the accuracies of the proposed HLD mechanism and other existing techniques [93,94,95] that also used data filtration along with feature extraction. In [93], the authors utilized a combination of IMU, mechanomyography, and electromyography sensors and filtered them using median, band-pass, and moving-average filters to remove noise. Next, they made windows of 5 s each from the data and applied different techniques for the feature extraction, including peak-to-peak, abrupt changes, skewness, and mean frequency. Further, to reduce the features’ vector dimension, they propose a multi-layer sequential forward selection method followed by classification via the random forest.

Haresamudram et al. present a self-supervised technique called masked reconstruction for HAR in [94]. They used small-labeled datasets and filtered data using transformer encoders. Then, they trained the network using different features and transfer learning mechanisms. In [95], a similar method to filter the data from motion, ambient, and vision-based sensors is proposed. The authors extracted features such as dynamic time warping, hidden Markov random fields, Mel-frequency cepstral coefficients, a gray-level co-variance matrix, and geodesic distance. Further, these features were optimized using a genetic algorithm and the system-recognized activities via a hidden Markov model-based classifier. As can be observed in Figure 16, the proposed HLD system with two novelties outperformed the existing works in terms of accuracy, sensitivity, and specificity.

### 4.5. Experiment 4: Comparisons with Existing Works

This section gives a comparison of our proposed IoHT-based HLD method with other previous state-of-the-art systems. We compared the proposed HLD system with methodologies that have hand-crafted descriptor extraction techniques, multiple datasets, machine learning, and applied deep learning techniques. Table 3 summarizes the comparison of the proposed system with other systems based on the classifiers, descriptor domain, modality, and accuracy achieved.

The comparison between multiple human activity recognition models is explained in the table. It focuses on the classifiers used to recognize these activities. The descriptors extracted for classification are also presented. Different models acquired either single- or multiple-sensor-based raw data. Single-sensor-based means that the data were acquired from one sensor type. In contrast, multimodal-sensor-based means that the data were gathered from multiple sensor types. The accuracies of each system compared are given in the table.

## 5. Discussion

**Figure 17:**
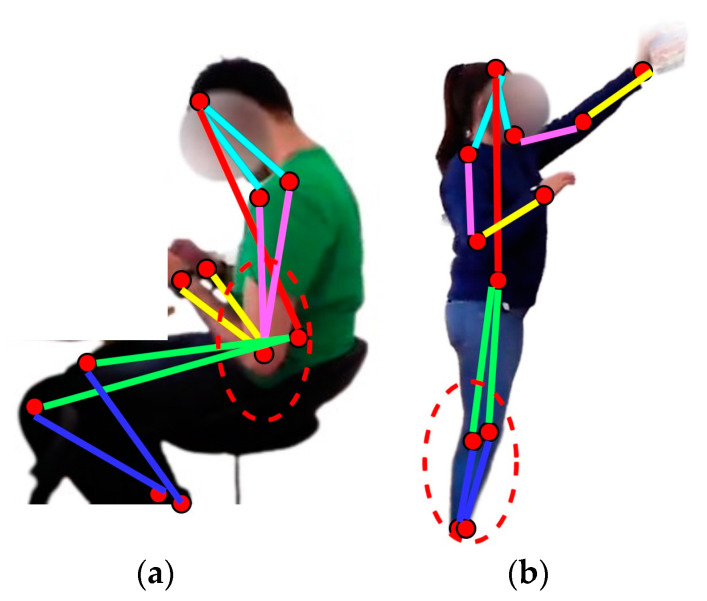
Samples of obstruction caused by human postures in activities over HWU-USP dataset: (**a**) using a phone and (**b**) taking out a bowl.

Although human locomotion decoding was achieved successfully using the proposed IoHT-based HLD system, this study also has a few limitations. The skeleton body points extracted can be obstructed in different human postures, which can cause limitations for accurate locomotion decoding. A couple of examples are highlighted in Figure 17 using red ellipses. The proposed filtration technique and descriptor extraction methodologies have to be assessed using some systems and datasets to verify the results. There is still a need to test this novel HLD system over different settings and datasets to validate the outcomes.

## 6. Conclusions

This article proposes a deep-learning-based human-locomotion-decoding system via novel filtration techniques and two innovative descriptor extraction mechanisms. The study compared two novelties of the proposed system using an RNN, a CNN, and LSTM. The RNN outperformed the other two deep learners concerning the accuracy of the IoHT-based HLD system. We have also shown that all the compared classifiers performed acceptably over the HWU-USP and LARa datasets. By comparing the three classifiers and other previous state-of-the-art methodologies, we conclude that the proposed IoHT-based HLD architecture enhances the accuracy rates for human locomotion decoding. Therefore, the proposed system has many applications in human activity decoding and can be scaled for more practical solutions in smart homes, ambient assisted living, and care-based facilities. In the future, we can compare and improve the results of the current study using different settings, datasets, and deep learning techniques.

## Figures and Tables

**Table 1:** Comparative analysis of proposed IoHT-based HLD system with other deep learning approaches using accuracy, recall, precision, and F1-score for the two benchmarked datasets.

Performance	Proposed System with First Novelty	Proposed System with Second Novelty	Proposed System with Both Novelties	CNN	LSTM
**HWU-USP**					
Accuracy	78.89%	80.00%	**82.22%**	72.22%	70.00%
Recall	0.79	0.80	**0.82**	0.72	0.70
Precision	0.79	0.81	**0.83**	0.73	0.71
*F*1-Score	0.79	0.81	**0.82**	0.72	0.70
**LARa**					
Accuracy	80.00%	77.50%	**82.50%**	78.75%	76.25%
Recall	0.80	0.77	**0.82**	0.78	0.76
Precision	0.80	0.78	**0.83**	0.79	0.76
*F*1-Score	0.80	0.77	**0.82**	0.79	0.76

**Table 2:** Confidence levels for skeleton body points over HWU-USP and LARa datasets.

Skeleton Body Points	Confidence Levels for HWU-USP	Confidence Levels for LARa
Head	0.95	0.94
Shoulders	0.92	0.90
Elbows	0.88	0.89
Wrists	0.91	0.90
Torso	0.85	0.88
Knees	0.89	0.92
Ankles	0.95	0.94
**Mean Confidence**	**0.90**	**0.91**

**Table 3:** Comparative analysis of proposed IoHT-based HLD system in terms of accuracy with existing work in the literature.

Ref.	Classifier	Descriptor Domain	Modality	Accuracy
[96]	Random Forest	Time-based	Multiple	81.00
[97]	CNN-LSTM	Deep-learning-based	Multiple	75.00
[98]	HMM	Machine learning	Single	78.33
[99]	Multi-Layer Perceptron	Frequency and time	Single	74.20
[100]	Multi-Layer Perceptron	Entropy	Multiple	75.50
[101]	Markov Chain	Multi-features	Multiple	74.94
[102]	Recurrent Neural Network	Convolutional	Multiple	82.00
[103]	Recurrent Neural Network	Raw	Single	80.43
**Proposed**	**Recurrent Neural Network**	**Energy, Graph, Frequency, and Time**	**Multiple**	**82.36**

## Data Availability

Data are contained within the article.
